# Complete mitochondrial genome of *Aphlugiolopsis trapeziformis* (Orthoptera: Tettigoniidae: Meconematinae)

**DOI:** 10.1128/mra.01113-25

**Published:** 2026-03-23

**Authors:** Jiaqi Huang, Yuqing Yao, Xun Bian, Bin Zhang

**Affiliations:** 1 College of Life Sciences and Technology, Inner Mongolia Normal University 71203 , Hohhot, China; 2 Guangxi Key Laboratory of Rare and Endangered Animal Ecology, Guangxi Normal University 12388, Guilin, China; 3 Key Laboratory of Biodiversity Conservation and Sustainable Utilization for College and University of Inner Mongolia Autonomous Region, Hohhot, China; University of California Riverside, Riverside, California, USA

**Keywords:** mitogenome, *Aphlugiolopsis*, Meconematinae, Orthoptera

## Abstract

In this study, we determined and analyzed the complete mitochondrial genome of *Aphlugiolopsis trapeziformis*. The mitogenome of *A. trapeziformis* (length: 16,766 bp) is double-stranded circular. It contains the 37 typical mitochondrial genes (13 protein genes, 22 tRNAs, and two rRNAs) and a large non-coding region, an A-T rich region.

## ANNOUNCEMENT

The *Aphlugiolopsis* is considered endemic to China, mainly distributed in southern China (1, 2), and currently comprises seven species (2, 3). Among them, the male individual of *Aphlugiolopsis trapeziformis* Cui & Bian, 2024 was first reported in Yunnan, China (4). It can be identified by a body that is yellowish brown, dorsal surface of the head with four longitudinal black-brown stripes, pronotum short with a blackish-brown longitudinal band, and tegmina not surpassing the apex of the abdomen (4). The molecular data obtained in this study will facilitate future studies on the identification, population genetics, and evolution of this genus.

The specimen of *A. trapeziformis* was collected in Tongbiguan, Yingjiang, Yunnan Province, China (24.608 N, 97.585 E). The voucher specimen was deposited in absolute anhydrous ethanol in the College of Life Sciences, Guangxi Normal University (GXNU). Total genomic DNA was extracted from the hind leg muscle tissue of the specimen using the TIANamp Genomic DNA Kit (TIANGEN) following the manufacturer’s instructions. Using the MGIEasy Kit (MGI), we constructed a paired-end library of 150-base pair and generated raw sequencing data on the Illumina NovaSeq 6000 (Illumina, Inc.) based on the sequencing platform’s principles. The raw data were processed with fastp v.0.20.0 (5) by trimming adapters and primers, filtering reads with phred quality < Q5, and filtering reads with N base number > 3. The sequencing generated 39,620,730 high-quality clean reads that were filtered. The complete mitochondrial genome was further assembled using the NOVOPlasty 4.3.3 (6) assembler, with the parameter configuration as follows: assembly type = mito, genome range = 14,000–18,000, k-mer size = 39, maximum memory = 16, and extended log = 0, and *Phlugiolopsis tuberculata* Xia & Liu, 1993 (7) as reference sequence (NC_068779) using CLC Genomics Workbench 12 with default parameters (8). The assembled sequence was annotated by MITOS 2.1.9 (9) webserver; start codon positions were confirmed and corresponded based on invertebrate mitochondrial genetic code and Refseq 89 Metazoa (10), with all other parameters following MITOS’ default settings. The mitogenomic map was depicted with Chloroplot 0.2.4 (11).

**Fig 1:**
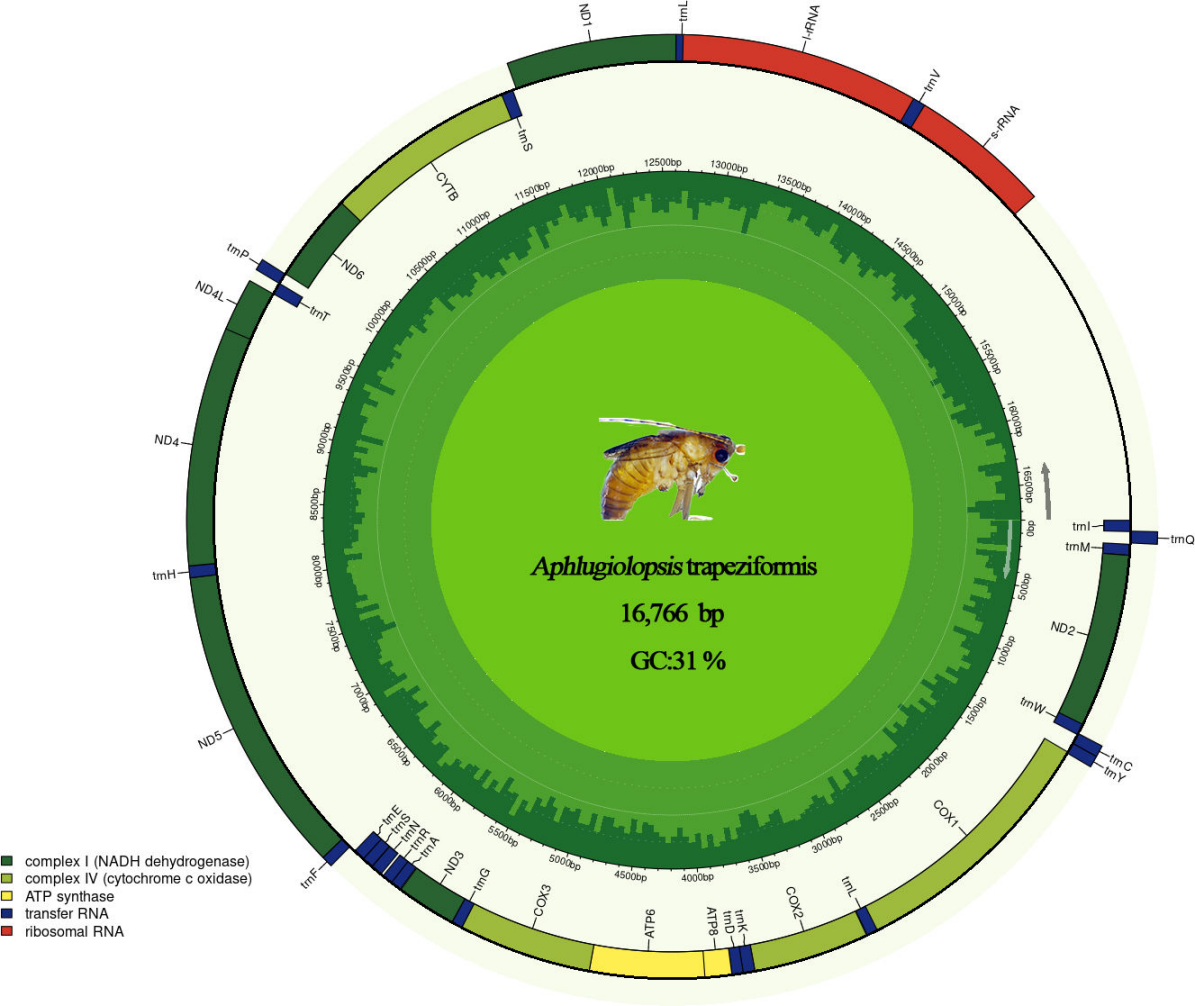
The circle map indicates the specimen’s total genome length and GC content. The innermost ring is labeled with GC content and transcription direction; the outermost ring shows the distribution of genes.

Sequencing results showed that the complete mitochondrial genome of this species is a closed circular double-stranded DNA molecule with a total length of 16,766 bp. Base composition analysis indicated that it has an adenine-thymine (AT) content of 69% and a guanine-cytosine (GC) content of 31%, exhibiting a typical AT-rich feature. The genome contains 37 standard mitochondrial genes and one non-coding control region (Fig. 1). In the mitochondrial genome, the start codons of all protein-coding genes follow the ATN rule, except for nad1 with TTG (Table 1). Among the 13 protein-coding genes, most terminate with TAA, three of which (ND1, ND3, and COB) terminate with TAG, and the rest of four genes (COX2, COX3, ND4, and ND5) are found to terminate with T (T stop codon is common in animal mitochondrial genomes and completed by the addition of 3′ A residues to the mRNA [12, 13]). The entire mitochondrial genome sequence of *A. trapeziformis* is 89% similar to the genome of *Phlugiolopsis tuberculata*.

**TABLE 1:** Gene annotation and genome structure analysis of *Aphlugiolopsis trapeziformis*

Gene	Type	Start	End	Length (bp)	Intergenic region length (bp)	Start/stop codons	Direction
tRNA-Ile	tRNA	1	67	67	0	–^a^	Forward
tRNA-Gln	tRNA	64	133	70	−4	–	Reverse
tRNA-Met	tRNA	141	205	65	7	–	Forward
nad2	CDS	206	1,234	1,029	0	ATT/TAA	Forward
tRNA-Trp	tRNA	1,232	1,299	68	−3	–	Forward
tRNA-Cys	tRNA	1,291	1,357	67	−9	–	Reverse
tRNA-Tyr	tRNA	1,357	1,422	66	−1	–	Reverse
cox1	CDS	1,415	2,959	1,545	−8	ATT/TAA	Forward
tRNA-Leu	tRNA	2,954	3,019	66	−6	–	Forward
cox2	CDS	3,023	3,707	685	3	ATG/T	Forward
tRNA-Lys	tRNA	3,707	3,777	71	−1	–	Forward
tRNA-Asp	tRNA	3,776	3,842	67	−2	–	Forward
atp8	CDS	3,843	4,007	165	0	ATT/TAA	Forward
atp6	CDS	4,001	4,678	678	−7	ATG/TAA	Forward
cox3	CDS	4,678	5,464	787	−1	ATG/T	Forward
tRNA-Gly	tRNA	5,464	5,528	65	−1	–	Forward
nad3	CDS	5,529	5,882	354	0	ATT/TAG	Forward
tRNA-Ala	tRNA	5,887	5,951	65	4	–	Forward
tRNA-Arg	tRNA	5,950	6,013	64	−2	–	Forward
tRNA-Asn	tRNA	6,029	6,096	68	15	–	Forward
tRNA-Ser	tRNA	6,098	6,165	68	1	–	Forward
tRNA-Glu	tRNA	6,165	6,232	68	−1	–	Forward
tRNA-Phe	tRNA	6,270	6,333	64	37	–	Reverse
nad5	CDS	6,334	8,065	1,732	0	ATT/T	Reverse
tRNA-His	tRNA	8,065	8,130	66	−1	–	Reverse
nad4	CDS	8,131	9,469	1,339	0	ATG/T	Reverse
nad4l	CDS	9,463	9,759	297	−7	ATG/TAA	Reverse
tRNA-Thr	tRNA	9,760	9,830	71	0	–	Forward
tRNA-Pro	tRNA	9,829	9,894	66	−2	–	Reverse
nad6	CDS	9,896	10,423	528	1	ATA/TAA	Forward
cob	CDS	10,423	11,559	1,137	−1	ATG/TAG	Forward
tRNA-Ser	tRNA	11,557	11,626	70	−3	–	Forward
nad1	CDS	11,649	12,596	948	22	TTG/TAG	Reverse
tRNA-Leu	tRNA	12,596	12,660	65	−1	–	Reverse
16sRNA	rRNA	12,635	13,965	1,331	−26	–	Reverse
tRNA-Val	tRNA	13,963	14,034	72	−3	–	Reverse
12sRNA	rRNA	14,033	14,821	789	−2	–	Reverse

^a^ –, not applicable.

## Data Availability

The complete mitochondrial genome sequence o*f Aphlugiolopsis trapeziformis* is available in GenBank under accession number PX412917. The associated BioProject, SRA, and BioSample numbers are PRJNA1335415, SRR35977086, and SAMN51987885, respectively.
